# Supplementary material for: Time-series transcriptome analysis identified differentially expressed genes in broiler chicken infected with mixed Eimeria species
Source: Front Genet. 2022 Aug 8;13:886781. doi: 10.3389/fgene.2022.886781 (PMC9393255; doi:10.3389/fgene.2022.886781)
Supplement: Supplementary file 2 [file DataSheet1.ZIP › 7dpi_KEGG.Gsea.1625115961950/KEGG_PHOSPHATIDYLINOSITOL_SIGNALING_SYSTEM.html]

Details for gene set KEGG\_PHOSPHATIDYLINOSITOL\_SIGNALING\_SYSTEM[GSEA]

|  || Dataset | TMM\_7dpi\_gct\_format\_7dpi\_gct\_format.Class\_4dpi.cls #PC\_versus\_NC.Class\_4dpi.cls #PC\_versus\_NC\_repos |
| Phenotype | Class\_4dpi.cls#PC\_versus\_NC\_repos |
| Upregulated in class | 0 |
| GeneSet | KEGG\_PHOSPHATIDYLINOSITOL\_SIGNALING\_SYSTEM |
| Enrichment Score (ES) | -0.49433997 |
| Normalized Enrichment Score (NES) | -1.6903288 |
| Nominal p-value | 6.551649E-4 |
| FDR q-value | 0.06355417 |
| FWER p-Value | 0.3106 |
Table: GSEA Results Summary

  

Fig 1: Enrichment plot: KEGG\_PHOSPHATIDYLINOSITOL\_SIGNALING\_SYSTEM      
 Profile of the Running ES Score & Positions of GeneSet Members on the Rank Ordered List

  

| SYMBOL | TITLE | RANK IN GENE LIST | RANK METRIC SCORE | RUNNING ES | CORE ENRICHMENT || 1 | PLCD4 | na | 176 | 1.084 | 0.0133 | No |
| 2 | PIK3C2B | na | 743 | 0.616 | -0.0182 | No |
| 3 | OCRL | na | 1555 | 0.366 | -0.0767 | No |
| 4 | PLCB4 | na | 2151 | 0.245 | -0.1201 | No |
| 5 | PLCD3 | na | 2185 | 0.238 | -0.1168 | No |
| 6 | INPP1 | na | 2880 | 0.141 | -0.1712 | No |
| 7 | PLCE1 | na | 2983 | 0.129 | -0.1764 | No |
| 8 | PLCD1 | na | 3024 | 0.123 | -0.1766 | No |
| 9 | PIP5K1C | na | 3125 | 0.111 | -0.1821 | No |
| 10 | PIP5K1B | na | 3713 | 0.039 | -0.2302 | No |
| 11 | DGKB | na | 4206 | -0.018 | -0.2710 | No |
| 12 | ITPR3 | na | 4900 | -0.092 | -0.3266 | No |
| 13 | DGKA | na | 5166 | -0.118 | -0.3457 | No |
| 14 | INPP5J | na | 5608 | -0.160 | -0.3785 | No |
| 15 | PI4KB | na | 5680 | -0.167 | -0.3801 | No |
| 16 | PIP5K1A | na | 5711 | -0.171 | -0.3783 | No |
| 17 | PIP4K2B | na | 5735 | -0.173 | -0.3757 | No |
| 18 | CDS2 | na | 5739 | -0.173 | -0.3715 | No |
| 19 | IMPA1 | na | 5761 | -0.177 | -0.3687 | No |
| 20 | PLCG1 | na | 6241 | -0.228 | -0.4029 | No |
| 21 | INPP5A | na | 6714 | -0.276 | -0.4353 | No |
| 22 | CDS1 | na | 6756 | -0.280 | -0.4315 | No |
| 23 | PLCB1 | na | 7317 | -0.343 | -0.4695 | No |
| 24 | CALM2 | na | 7385 | -0.350 | -0.4661 | No |
| 25 | INPP5E | na | 7472 | -0.358 | -0.4640 | No |
| 26 | DGKH | na | 7586 | -0.370 | -0.4639 | No |
| 27 | ITPR1 | na | 7928 | -0.411 | -0.4819 | No |
| 28 | INPP5K | na | 8078 | -0.432 | -0.4832 | Yes |
| 29 | CALM1 | na | 8103 | -0.435 | -0.4740 | Yes |
| 30 | SYNJ2 | na | 8110 | -0.436 | -0.4632 | Yes |
| 31 | ITPKA | na | 8252 | -0.453 | -0.4633 | Yes |
| 32 | PIK3CG | na | 8478 | -0.481 | -0.4698 | Yes |
| 33 | PTEN | na | 8620 | -0.503 | -0.4686 | Yes |
| 34 | PRKCA | na | 8704 | -0.513 | -0.4623 | Yes |
| 35 | IMPA2 | na | 9082 | -0.562 | -0.4793 | Yes |
| 36 | DGKZ | na | 9155 | -0.574 | -0.4705 | Yes |
| 37 | IPPK | na | 9226 | -0.587 | -0.4612 | Yes |
| 38 | DGKG | na | 9451 | -0.624 | -0.4639 | Yes |
| 39 | INPPL1 | na | 9459 | -0.624 | -0.4484 | Yes |
| 40 | INPP5B | na | 9519 | -0.634 | -0.4369 | Yes |
| 41 | PIK3R2 | na | 9597 | -0.645 | -0.4267 | Yes |
| 42 | PIP4K2A | na | 9723 | -0.664 | -0.4201 | Yes |
| 43 | PI4KA | na | 9785 | -0.671 | -0.4078 | Yes |
| 44 | PRKCB | na | 9867 | -0.685 | -0.3969 | Yes |
| 45 | PIK3C2G | na | 10128 | -0.737 | -0.3997 | Yes |
| 46 | PIK3CB | na | 10335 | -0.777 | -0.3968 | Yes |
| 47 | PLCG2 | na | 10697 | -0.853 | -0.4050 | Yes |
| 48 | PIK3CD | na | 10707 | -0.855 | -0.3837 | Yes |
| 49 | PLCB2 | na | 10772 | -0.872 | -0.3666 | Yes |
| 50 | PIK3CA | na | 10920 | -0.912 | -0.3553 | Yes |
| 51 | PIK3R5 | na | 10941 | -0.918 | -0.3333 | Yes |
| 52 | PIK3R3 | na | 11045 | -0.949 | -0.3174 | Yes |
| 53 | PIK3C3 | na | 11107 | -0.966 | -0.2976 | Yes |
| 54 | INPP4A | na | 11216 | -1.005 | -0.2806 | Yes |
| 55 | PIK3C2A | na | 11243 | -1.015 | -0.2566 | Yes |
| 56 | PIK3R1 | na | 11265 | -1.023 | -0.2320 | Yes |
| 57 | DGKQ | na | 11364 | -1.064 | -0.2127 | Yes |
| 58 | SYNJ1 | na | 11396 | -1.079 | -0.1874 | Yes |
| 59 | INPP5D | na | 11445 | -1.096 | -0.1632 | Yes |
| 60 | DGKE | na | 11540 | -1.132 | -0.1418 | Yes |
| 61 | PIKFYVE | na | 11699 | -1.238 | -0.1231 | Yes |
| 62 | ITPR2 | na | 11767 | -1.303 | -0.0950 | Yes |
| 63 | INPP4B | na | 11768 | -1.304 | -0.0613 | Yes |
| 64 | ITPK1 | na | 11837 | -1.390 | -0.0311 | Yes |
| 65 | DGKD | na | 11969 | -1.764 | 0.0034 | Yes |
Table: GSEA details [plain text format]

  

Fig 2: KEGG\_PHOSPHATIDYLINOSITOL\_SIGNALING\_SYSTEM      
 Blue-Pink O' Gram in the Space of the Analyzed GeneSet

  

Fig 3: KEGG\_PHOSPHATIDYLINOSITOL\_SIGNALING\_SYSTEM: Random ES distribution      
 Gene set null distribution of ES for **KEGG\_PHOSPHATIDYLINOSITOL\_SIGNALING\_SYSTEM**
